# Comprehensive analysis of allergen-specific IgE in COPD: mite-specific IgE specifically related to the diagnosis of asthma-COPD overlap

**DOI:** 10.1186/s13223-021-00514-9

**Published:** 2021-02-04

**Authors:** Hikaru Toyota, Naoya Sugimoto, Konomi Kobayashi, Yuki Suzuki, Yuri Takeshita, Ayaka Ito, Mariko Ujino, Fuminori Tomyo, Hirokazu Sakasegawa, Yuta Koizumi, Michio Kuramochi, Masao Yamaguchi, Hiroyuki Nagase

**Affiliations:** Division of Respiratory Medicine and Allergology, Department of Medicine, Teikyo University School of Medicine, 2-11-1, Kaga, Itabashi-ku, Tokyo, 173-8605 Japan

**Keywords:** ACO, Cockroach, *Dermatophagoides pteronyssinus*, Eosinophils, Fraction of exhaled nitric oxide, View allergy 39

## Abstract

**Background:**

Although the relationship between allergic sensitization and increased respiratory symptoms of chronic obstructive pulmonary disease (COPD) has been suggested, which allergen has a significant effect on COPD pathology is unclear. This study aimed to identify the specific IgE related to clinical features of COPD and the diagnosis of asthma-COPD overlap (ACO).

**Methods:**

We recruited 76 patients with COPD and analyzed 39 IgE using panel IgE test (View Allergy 39^®^). ACO was diagnosed according to the Japanese Respiratory Society Guidelines.

**Results:**

As for perennial aeroallergens, the positivity for moth (31.5%), *Candida* (23.7%), *Dermatophagoides pteronyssinus* (22.4%) and house dust (22.4%), and concerning pollen, Japanese cedar (35.5%) and Japanese cypress (22.2%) exceeded 20%. Only the positivity of IgE for *Dermatophagoides pteronyssinus* and house dust was significantly higher in ACO compared with that in non-ACO COPD. Moreover, it contributed to the diagnosis of ACO in an IgE class-dependent manner. Patients with cockroach IgE exhibited higher residual volume, whereas those with Japanese cedar IgE exhibited better diffusion capacity than negative patients. The contribution for ACO diagnosis by the receiver operating characteristic curve analysis was comparable among total IgE (cutoff value: 158 IU/mL), blood eosinophil count (234/μL), and fraction of exhaled nitric oxide (31.0 ppb).

**Conclusions:**

The prominent role of mite-specific IgE in the diagnosis and pathology of ACO and the potentially detrimental effect of cockroach sensitization on air trapping in COPD were suggested. The finding highlights the future development of a treatment targeting IgE as a treatable trait in COPD.

## Background

Allergic sensitization is an established feature of asthma, and assessment of sensitization status is recommended for patients with asthma [1]. In contrast, allergic sensitization may not be routinely assessed in patients with chronic obstructive pulmonary disease (COPD), and the diagnosis of allergy is not recommended by the Global Initiative for Chronic Obstructive Lung Disease (GOLD) [2]. However, allergic sensitization has been reported to be related to increased respiratory symptoms and risk of exacerbations in patients with COPD [3] and may be a potential treatable trait by avoidance of allergen or by anti-immunoglobulin E (IgE) treatment [4]. Although the positivity of 5 [3] or 6 [5] allergen-specific IgE in patients with COPD was analyzed, the number of tested allergens was limited, and the relationship between allergen-specific IgE and patient characteristics was not fully elucidated.

The concept of asthma-COPD overlap (ACO) has been introduced in recent years, and various diagnostic criteria have been proposed [6]. Allergic sensitization leads to type 2 inflammation, which is a prominent feature of asthma and may serve as an important component of ACO diagnosis. However, the diagnostic criteria of ACO did not always include IgE categories [7]. Although some recent guidelines introduced elevated levels of IgE into the diagnostic criteria [8, 9], the cutoff level of IgE was not stated in the guidelines, including the Japanese Respiratory Society (JRS) published in 2018 [10, 11]. Some studies compared the levels of total and several allergen-specific IgE between ACO and pure COPD and revealed that the levels of total IgE [5] or *Dermatophagoides*-specific IgE [5, 6] were higher in ACO. However, the number of tested allergens was limited, and which allergen-specific IgE is optimal for ACO diagnosis remains unclear. Concerning JRS guideline, although the prevalence of ACO was reported to be 30.4% among patients with fixed airflow limitation [10], the positivity of specific IgE in ACO was not independently exhibited [10]. In addition, although recent evidence suggests the interactions between food allergy and asthma [12], the relationship between food allergy and ACO or COPD is largely unknown.

This study aimed to comprehensively analyze the panel of specific IgE among patients with COPD and to investigate the relationship between each specific IgE and clinical features, including pulmonary function, or ACO diagnosis. To do this, we adopted panel IgE test that can simultaneously measure 39 specific IgE. In addition, we compared the contribution of IgE with the fraction of exhaled nitric oxide (FeNO) or blood eosinophils with ACO diagnosis and attempted to elucidate the relative importance of IgE.

## Methods

### Study design and patient population

This was a cross-sectional study that investigated COPD patients in the outpatient clinic of Teikyo University Hospital. COPD was diagnosed according to the GOLD criteria [2]. Medical information and laboratory findings were retrospectively obtained from medical records and estimated under stable conditions before completing the survey for ACO. Specific IgE levels were measured using panel IgE test (View Allergy 39^®^, Thermo Fisher Diagnostics K.K., Tokyo, Japan) and judged as positive when the class was equal to or greater than 1. NIOX VERO^®^ (Aerocrine, Morrisville, NC, USA) was used to measure FeNO. The panel IgE test includes specific IgE for pollens or molds, which are shown to be highly prevalent in Japan [13].

### Definition of ACO

ACO was diagnosed according to the JRS Guidelines for the Management of ACO 2018 [11]. In brief, ACO was defined as having fixed airflow limitation (forced expiratory volume in 1 s [FEV_1_]/forced vital capacity [FVC] < 70%) with 1 or more of the COPD features plus 2 or more of the asthma features (Additional File 1). Variable or paroxysmal symptoms suggesting asthma were analyzed by self-completed questionnaire. The diagnosis of perennial rhinitis was made by self-completed SACRA questionnaire based on the Allergic Rhinitis and Its Impact on Asthma guidelines [14]. The criterion for elevated IgE was judged as positive when the level of serum total IgE exceeded 100 IU/mL based on the institutional standard value or when the class of specific IgE was equal to or greater than class 1. Although we analyzed 85 COPD patients (Additional File 2), a portion of the patients could not be judged to have ACO or not because all tests indicated in Additional File 1 could not be performed. As such, these patients were excluded from the analysis concerning ACO. Among evaluable 76 patients, 44.7% of the patients were diagnosed with ACO.

### Statistical analysis

Bartlett’s test was employed to check the variances across samples. Differences between the two groups were analyzed using Student’s t-test or Mann–Whitney U-test when the variances were equal or different, respectively. The relationship between biomarkers was analyzed by Spearman’s correlation coefficient. The receiver operating characteristic (ROC) analysis was employed to find a cutoff value for biomarkers to detect ACO diagnosis. Multivariate logistic regression analysis was employed to estimate odds ratios (ORs) with 95% confidence intervals (CIs) for ACO diagnosis. Statistical significance was defined as *P* < 0.05, and data were presented as mean ± standard error of the mean. JMP^®^ 14 (SAS Institute Japan, Tokyo, Japan) or Prism^®^ 8 (GraphPad Software, San Diego, CA, USA) was used for statistical analyses.

## Results

**Fig. 1:**
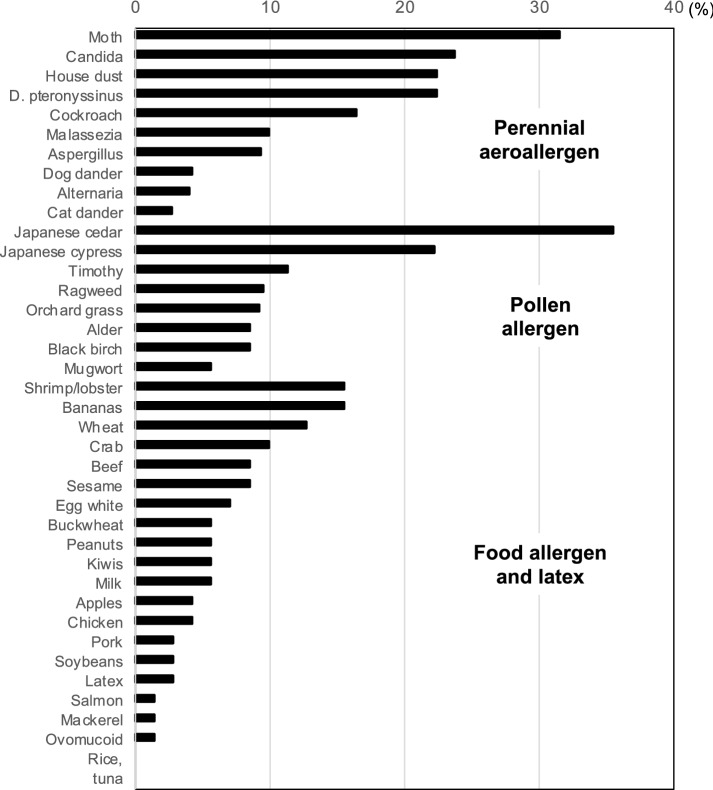
The positivity of specific immunoglobulin E (IgE) in chronic obstructive pulmonary disease. Specific IgE levels were judged as positive when the class was equal to or greater than 1

We analyzed the positivity of 39 allergen-specific IgE in all COPD patients. Concerning the perennial aeroallergens, the positivity for moth (31.5%), *Candida* (23.7%), *Dermatophagoides pteronyssinus* (*D. pteronyssinus*, 22.4%), and house dust (22.4%) was > 20% (Fig. 1). As for pollen allergens, the positivity for Japanese cedar (35.5%) and Japanese cypress (22.2%) exceeded 20%. As for foods or other allergens, the positivity for shrimp/lobster (15.5%), bananas (15.5%), and wheat (12.7%) was higher than 10%.

**Fig. 2:**
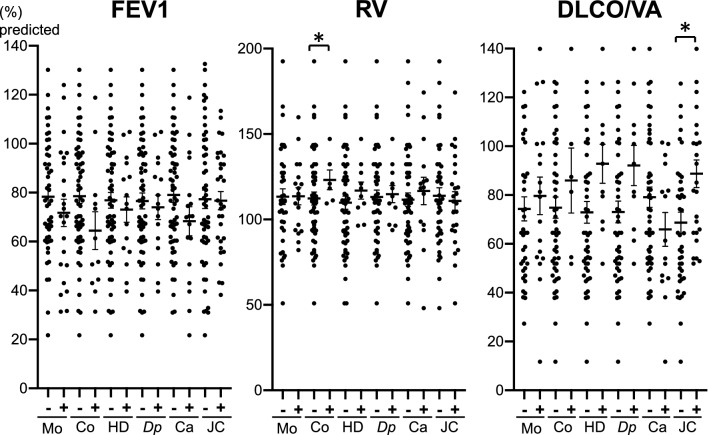
The comparison of pulmonary function between specific immunoglobulin E. (IgE)-positive and specific IgE-negative patients with chronic obstructive pulmonary disease. Specific IgE levels were judged as positive when the class was equal to or greater than 1. *P* < 0.05* between specific IgE positive and specific IgE-negative group. *Ca* Candida, *Co* cockroach, *DP* *Dermatophagoides pteronyssinus*, *DLco/V*_*A*_ diffusion capacity of carbon monoxide/alveolar volume, *FEV*_*1*_ forced expiratory volume in 1 s, *HD* house dust, *JC* Japanese cedar, *Mo* moth, *RV* residual volume

We also compared the characteristics of patients who are positive with those who are negative for specific IgE (Fig. 2; Additional File 3). In patients with positive IgE for cockroach, residual volume (RV) was significantly higher (136.7% vs 110.2%), and FEV_1_ tended to be lower (69.5% vs 78.7%) compared with patients with negative IgE (Fig. 2). Contrarily, in patients with positive IgE for Japanese cedar, the diffusion capacity of carbon monoxide/alveolar volume (DL_CO_/V_A_) was significantly higher than in patients with negative IgE (92.1% vs 73.2%) (Fig. 2). The prevalence of perennial allergic rhinitis was significantly higher in patients with positive specific IgE for all allergens, except for Japanese cedar, which causes seasonal rhinitis only in the spring season (Additional File 3).

Furthermore, we compared patient characteristics between ACO and non-ACO COPD (Table 1). In ACO, body mass index and FeNO were significantly higher compared with non-ACO COPD. FEV_1_, %predicted also tended to be lower in ACO, and FeNO was significantly higher in patients with values that fell in the lower half of FEV_1_, %predicted (26.3 vs 40.8 ppb, *P* < 0.05). A significantly greater proportion of patients with ACO exhibited all the features of asthma, except for airway reversibility (Additional File 4).

**Table 1:** Comparison of patient characteristics between ACO and non-ACO COPD patients

	ACO	Non-ACO COPD	Total	*P* value
Subject (n)	34	42	76	
Sex male (%)	82.4	81.0	81.6	0.88
Body mass index (kg/m^2^)	24.1 ± 0.6	22.3 ± 0.6	23.1 ± 3.8	0.042*
Age (years)	72.5 ± 1.3	74.3 ± 1.2	73.5 ± 7.6	0.30
Smoking status (current/ex/never [%])	20.6/79.4/0	7.1/90.5/2.4	13.2/85.5/1.3	0.16
Smoking history (pack-years)	55.7 ± 5.8	62.0 ± 5.2	59.2 ± 34.0	0.43
FVC, %predicted	107.6 ± 3.1	105.4 ± 2.8	106.4 ± 17.8	0.59
FEV_1_ (L)	1.68 ± 0.12	1.82 ± 0.10	1.76 ± 0.68	0.39
FEV_1_, %predicted	72.9 ± 4.1	79.6 ± 3.7	76.6 ± 24.2	0.24
FEV_1_/FVC ratio (%)	51.5 ± 2.4	57.1 ± 2.2	54.6 ± 14.1	0.088
RV, % predicted^a^	111.2 ± 5.1	112.8 ± 5.0	112.0 ± 27.2	0.82
DLco/V_A_, % predicted^b^	80.7 ± 5.7	72.6 ± 5.6	76.6 ± 30.9	0.32
Airway reversibility (mL)^c^	131.0 ± 23.1	102.9 ± 21.6	116.0 ± 105.6	0.38
Airway reversibility (%)^c^	9.3 ± 1.8	5.8 ± 1.7	7.4 ± 8.4	0.16
Blood eosinophil count (cells/μL)	248 ± 29	218 ± 26	231 ± 167	0.45
Serum total IgE (IU/mL)	375 ± 84	258 ± 75	310 ± 488	0.31
FeNO (ppb)^d^	33.8 ± 2.6	20.7 ± 2.3	26.4 ± 16.1	< 0.01**
ICS use before diagnosis of ACO (%)	35.3	7.1	19.7	< 0.01**
LABA (%)	85.3	73.8	79.0	0.22
LAMA (%)	82.4	81.0	81.6	0.88
Theophylline (%)	20.6	14.3	17.1	0.47
LTRA (%)	17.7	2.4	9.2	0.022*
Antihistamine (%)	14.7	7.1	10.5	0.29

*ACO* asthma-COPD overlap, *COPD* chronic obstructive pulmonary disease, *DLco/V*_*A*_ diffusing capacity of carbon monoxide/alveolar volume, *FeNO* fraction of exhaled nitric oxide, *FEV*_*1*_ forced expiratory volume in 1 s, *FVC* forced vital capacity, *ICS* inhaled corticosteroid, *IgE* immunoglobulin E, *LABA* long-acting β-agonists, *LAMA* long-acting muscarinic receptor antagonist, *RV* residual volume, *LTRA* leukotriene receptor antagonist

^a^RV

^b^DLco/V_A_ (ACO n = 29, non-ACO COPD n = 30)

^c^Airway reversibility (ACO n = 21, non-ACO COPD n = 24)

^d^FeNO (ACO n = 33, non-ACO COPD n = 42)

*P* < 0.05*, *P* < 0.01** between ACO and non-ACO COPD groups

**Fig. 3:**
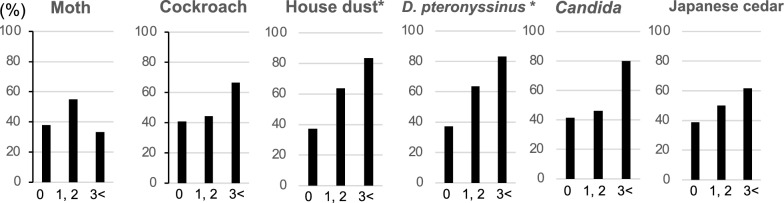
The relationship between the value of immunoglobulin E (IgE) class and the proportion of patients diagnosed with asthma-COPD overlap. *P* < 0.05* indicates significant difference between groups. *D. pteronyssinus, Dermatophagoides pteronyssinus*

Next, we compared the positivity of specific IgE between ACO and non-ACO COPD (Table 2). Among various aeroallergens, only the positivity for house dust and *D. pteronyssinus* was significantly higher in ACO. As for other perennial aeroallergens, the positivity for moth, *Candida*, and *Malassezia* was numerically higher in ACO without statistical significance. As for pollen, the positivity for Japanese cedar tended to be higher in ACO (*P* = 0.16). Concerning food allergens, the overall positivity for beef-specific IgE was low (8.5%); however, the positivity was significantly lower in ACO. We also analyzed the relationship between the value of IgE class and the proportion of patients diagnosed with ACO (Fig. 3). Only house dust- and *D. pteronyssinus*-specific IgE showed significant relationship between the class of specific IgE and ACO diagnosis.

**Table 2:** Comparison of positivity in specific IgE between ACO and non-ACO COPD

	ACO	Non-ACO COPD	Total	*P *value		ACO	Non-ACO COPD	Total	*P *value
Perennial aeroallergen					Food allergen and latex				
Moth	38.7	26.2	31.5	0.26	Shrimp/lobster	16.7	14.6	15.5	0.82
*Candida*	29.4	19.1	23.7	0.29	Bananas	20.0	12.2	15.5	0.37
House dust	35.3	11.9	22.4	0.015**	Wheat	13.3	12.2	12.7	0.89
*D. pteronyssinus*	35.3	11.9	22.4	0.015**	Crab	3.3	14.6	9.9	0.11
Cockroach	19.4	14.3	16.4	0.56	Beef	0.0	14.6	8.5	0.029*
*Malassezia*	13.3	7.3	9.9	0.40	Sesame	3.3	12.2	8.5	0.18
*Aspergillus*	12.1	7.1	9.3	0.46	Buckwheat	3.3	7.3	5.6	0.47
Dog dander	3.3	4.8	4.2	0.76	Apples	0.0	7.3	4.2	0.13
*Alternaria*	6.1	2.4	4.0	0.42	Chicken	0.0	7.3	4.2	0.13
Cat dander	6.1	0.0	2.7	0.11	Egg white	6.7	7.3	7.0	0.92
Pollen allergen					Peanuts	3.3	7.3	5.6	0.47
Japanese cedar	44.1	28.6	35.5	0.16	Kiwis	6.7	4.9	5.6	0.75
Japanese cypress	19.4	24.4	22.2	0.61	Milk	3.3	7.3	5.6	0.47
Orchard grass	14.7	9.5	11.8	0.49	Pork	0.0	4.9	2.8	0.22
Timothy	13.3	9.8	11.3	0.64	Soybeans	0.0	4.9	2.8	0.22
Ragweed	11.8	7.1	9.2	0.49	Latex	0.0	4.9	2.8	0.22
Alder	3.3	12.2	8.5	0.18	Salmon	0.0	2.4	1.4	0.39
Black birch	3.3	12.2	8.5	0.18	Mackerel	0.0	2.4	1.4	0.39
Mugwort	3.3	7.3	5.6	0.47	Ovomucoid	3.3	0.0	1.4	0.24
					Rice, tuna	0.0	0.0	0.0	−

n = 76, *P* < 0.05*, *P* < 0.01** between ACO and non-ACO COPD groups. Specific IgE was judged as positive when the class was equal to or greater than 1

*ACO* asthma-COPD overlap, *COPD* chronic obstructive pulmonary disease, *D. pteronyssinus* *Dermatophagoides pteronyssinus*

**Fig. 4:**
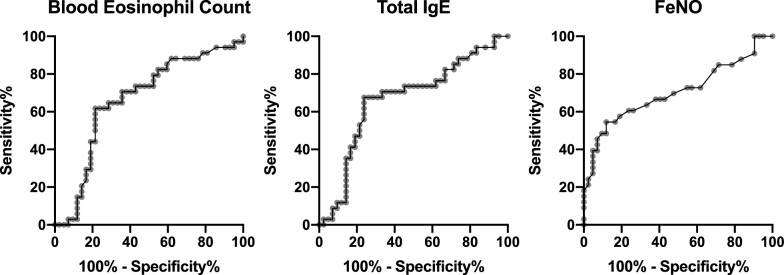
The receiver operating characteristic analysis of biomarkers to detect the diagnosis of asthma-COPD overlap. The area under the curve was 0.666 for blood eosinophil count, 0.665 for total immunoglobulin E (IgE), and 0.703 for fraction of exhaled nitric oxide (FeNO). The best cutoff value was 234 count/μL for blood eosinophils (sensitivity 0.618, specificity 0.786), 158 IU/mL for serum total IgE (sensitivity 0.677, specificity 0.662), and 31.0 ppb for FeNO (sensitivity 0.546, specificity 0.881)

As the diagnostic criteria for ACO included FeNO and blood eosinophil count, in addition to IgE levels, we compared the utility of those biomarkers in ACO diagnosis by the ROC curve analysis. Although there were weak or no significant relationships between those biomarkers (Additional File 5), the area under the curve for ACO diagnosis was comparable among three biomarkers: 0.666 (95% CI 0.540–0.793, *P* = 0.013) for blood eosinophil count, 0.665 (95% CI 0.538–0.791, *P* = 0.014) for total IgE, and 0.703 (95% CI 0.578–0.829, *P* = 0.0027) for FeNO (Fig. 4). There were no significant differences between the ROC curves for ACO diagnosis (IgE vs blood eosinophil count: *P* = 0.99, IgE vs FeNO *P* = 0.67, FeNO vs blood eosinophil count: *P* = 0.64). The best cutoff value for diagnosis was 234 count/μL for blood eosinophils, 158 IU/mL for serum total IgE, and 31.0 ppb for FeNO.

The contribution of blood eosinophils, FeNO, and IgE was analyzed to further determine the relative importance of the different biomarkers in ACO diagnosis. In univariate analysis, the criteria, except for blood eosinophils, significantly contributed to ACO diagnosis (FeNO: OR 9.58, 95% CI 2.45–37.4, *P* < 0.01, IgE: OR 3.93, 95% CI 1.45–10.68, *P* < 0.01). In addition, although specific IgE for house dust (*P* = 0.0146) and *D. pteronyssinus* (*P* = 0.0146) significantly contributed to the diagnosis of ACO, positivity for moth (*P* = 0.2564), cockroach (*P* = 0.5653), Japanese cedar pollen (*P* = 0.1593), and *Candida* (*P* = 0.2917) did not exhibit significant contributions. We also analyzed the contribution of the number of sensitized allergens, and the number did not contribute to the diagnosis of ACO (*P* = 0.98).

Concerning IgE factors, all of the following criteria significantly contributed to ACO diagnosis: total IgE > 100 IU/mL, positive for house dust-specific IgE, and positive for *D. pteronyssinus*-specific IgE. In the three models for multivariate analyses (Table 3), all IgE criteria significantly contributed to ACO diagnosis, in addition to FeNO > 35 ppb criterion.

**Table 3:** Multivariate analysis of biomarkers for the diagnosis of ACO

	OR	CI		*P* value
Model 1				
Blood eosinophils > 300 μL	1.25	0.39	4.02	0.71
FeNO > 35 ppb	15.34	3.03	77.57	< 0.01**
IgE > 100 IU/mL	6.18	1.74	21.88	< 0.01**
Model 2				
Blood eosinophils > 300 μL	2.12	0.69	6.45	0.19
FeNO > 35 ppb	7.98	1.95	32.74	< 0.01**
House dust IgE positive	3.74	1.04	13.46	0.038*
Model 3				
Blood eosinophils > 300 μL	2.12	0.70	6.45	0.19
FeNO > 35 ppb	7.98	1.95	32.74	< 0.01**
* D. pteronyssinus* IgE positive	3.74	1.04	13.46	0.038*

Specific IgE was judged as positive when the class was equal to or greater than 1. The sensitivity, specificity, positive predictive value, and negative predictive value for diagnosis of ACO are as follows: Blood eosinophils > 300 μL (44.1%, 76.2%, 60.0%, 62.7%), FeNO > 35 ppb (42.4%, 92.9%, 82.4%, 67.2%), IgE > 100 IU/mL (70.6%, 61.9%, 60.0%, 72.2%), House dust IgE positive (35.3%, 88.1%, 70.6%, 62.7%)*, D. pteronyssinus* IgE positive (35.3%, 88.1%, 70.6%, 62.7%)

*CI* confidence interval, *D. pteronyssinus* *Dermatophagoides pteronyssinus*, *FeNO* fraction of exhaled nitric oxide, *IgE* immunoglobulin E, *OR* odds ratios

## Discussion

For the first time, we comprehensively analyzed a panel of 39 specific IgE in patients with COPD and revealed the importance of *D. pteronyssinus*—and house dust-specific IgE for the diagnosis or pathology of ACO by the following observations. First, the positivity of *D. pteronyssinus* and house dust was significantly higher in ACO group than in the non-ACO COPD group (Table 2). Second, the frequency of ACO diagnosis was increased in IgE class-dependent manner of those allergens (Fig. 3). Lastly, multivariate analysis revealed the significant contribution of total IgE and house dust- and *D. pteronyssinus-*specific IgE to ACO diagnosis (Table 3). As mites have been identified as a major antigenic substance in house dust [15], those results suggested the specifically important role of sensitization to mite allergen in the pathology or diagnosis of ACO. In addition, as the patients with cockroach-specific IgE exhibited higher RV and patients with Japanese cedar-specific IgE exhibited better DL_CO_/V_A_ compared with negative patients (Fig. 2), the allergen-specific effect on COPD pathology was also suggested.

A previous report using skin prick test or ImmunoCAP, wherein results were highly correlated with the current panel IgE test [16], revealed that the positivity of *D. pteronyssinus*—and house dust-specific IgE in patients with asthma was higher compared with patients with COPD [17, 18]. Contrarily, the positivity for *Candida* (23.7%) and moth (31.5%) in this study was comparable to the reported positivity in asthma [17] and was not significantly different between ACO and non-ACO COPD (Table 2). Those results suggested the specifically important role of sensitization to mites in the diagnosis of ACO from COPD.

The positivity for cockroach-specific IgE in COPD has been reported to be comparable to the positivity in asthma, but higher than in the control group [13, 18, 19]. In addition, no significant difference in positivity of cockroach-specific IgE was observed between ACO and non-ACO COPD in this study (Table 1). Taken together, sensitization to cockroach seems to have little role in distinguishing asthma and COPD or diagnosing ACO. Interestingly, RV was significantly higher, and the FEV_1_ tended to be lower in cockroach IgE positive COPD (Fig. 2). The high level of exposure to cockroach was reported to increase the risk of asthma development [20] and its severity [21]. Cockroach extract was shown to induce secretions of various cytokines and chemokines from airway epithelial cells [22, 23]. In addition, allergen component of cockroach, Per a 10 is a serine protease, which induces the generation of IL-6, IL-8, and GM-CSF from epithelial cells through the activation of protease-activated receptor 2 [24, 25]. These proinflammatory natures of cockroach allergen might cause a detrimental effect on the airway structure of COPD, and detection of cockroach-specific IgE might help identify air trapping in COPD.

The reason why DL_CO_/V_A_ was significantly higher in Japanese cedar-sensitized patients (Fig. 2) remains unclear. Japanese cedar pollen is the most common seasonal allergen in Japan and causes seasonal allergic rhinitis during the spring season. Concomitant pollinosis was not related to chronic asthma severity, although asthma exacerbations increased during the cedar pollen season [26]. As cedar pollens have a diameter of more than 30 μm, they mainly deposit in the upper airways and might not have a detrimental effect on gas exchange.

Recognizing treatable traits in chronic airway diseases has been emphasized [4], and the definition of ACO was not referred in the latest GOLD document [2]. Nevertheless, the cluster analysis based on transcription factor characterizing the subset of helper T cells identified the ACO phenotype with elevated IgE and blood eosinophils, suggesting the existence of ACO endotypes [27]. In addition, detecting specific IgE in COPD is still important, because IgE-mediated inflammation is potentially a treatable trait. The importance of sensitized allergen avoidance has been stressed [4], and the effect of anti-IgE antibody, omalizumab, is expected in ACO. A small retrospective analysis of omalizumab revealed the improvement in the symptoms of patients with severe asthma who have smoking history and fixed airway obstruction [28]. However, studies on ACO are limited [29, 30], and another exploratory study of omalizumab in patients with COPD with elevated IgE level was terminated due to difficulty in recruiting eligible patients (NCT00851370). In our analysis, patients diagnosed with ACO tended to show lower FEV_1_ compared with non-ACO COPD, and patients with cockroach-specific IgE exhibited higher RV than nonsensitized patients. In this context, the efficacy of anti-IgE treatment for ACO diagnosed from clinical COPD or COPD sensitized with cockroach should be established in the future study.

This study has some limitations. The importance of IgE for ACO diagnosis may vary depending on the diagnostic criteria. There are various diagnostic criteria for ACO including [8, 11] or not including IgE [5, 7, 9]. When we applied the consensus definition by Sin et al. [9] to our study population, the proportion of patients with IgE > 100 IU/mL or positive IgE for *D. pteronyssinus* tended to be higher in ACO than in non-ACO COPD (68.8% vs 48.0% [*P* = 0.148] and 37.5% vs 20.0% [*P* = 0.155], respectively)*.* Although the use of ICS may affect the diagnosis of ACO, only three patients were treated by ICS in non-ACO COPD; therefore, the effect on the overall results was limited. Due to the nature of multiple testing, analyzing more patients is desirable for future analysis especially concerning the role of specific IgE for pollens and cockroach. In addition to our current findings, the general importance of mite-specific IgE in the diagnosis or pathology of ACO was suggested. Although geographical variation in the positivity of specific IgE may exist, sensitization to *D. pteronyssinus* was most prevalent among various developed countries [31] and different areas in Japan [13]. It remains unclear whether ACO diagnosed from clinical asthma and clinical COPD has identical pathophysiology. Our findings concerning the utility of IgE for diagnosing ACO is limited to ACO diagnosed from COPD.

## Conclusions

In conclusion, by analyzing a panel of specific IgE in COPD, we revealed the specifically important role of mite-specific IgE in the diagnosis or pathology of ACO and the potentially detrimental effect of cockroach sensitization to air trapping in COPD. The finding highlights future study for developing a novel treatment strategy targeting IgE as a treatable trait in patients with ACO.

## Supplementary Information

**Additional file 1** Features of COPD or asthma described by the Japanese Respiratory Society Guideline

**Additional file 2** The number of patients analyzed in the study

**Additional file 3** Comparison of patient characteristics based on positivity for allergen specific IgE

**Additional file 4** Proportion of patients fulfilling the criteria for features of asthma

**Additional file 5** Relationships between blood eosinophil count, total IgE level, and FeNO

## Data Availability

The datasets used and/or analysed during the current study are available from the corresponding author (nagaseh@med.teikyo-u.ac.jp) on reasonable request under permission from Ethical Review Board.

## Abbreviations

ACO: Asthma-COPD overlap
CI: Confidence intervals
COPD: Chronic obstructive pulmonary disease
DL_CO_/V_A_: Diffusing capacity of carbon monoxide/alveolar volume
*D. pteronyssinus*: *Dermatophagoides pteronyssinus*
FeNO: Fraction of exhaled nitric oxide
FEV_1_: Forced expiratory volume in 1 s
FVC: Forced vital capacity
GOLD: Global initiative for chronic obstructive lung disease
IgE: Immunoglobulin E
JRS: Japanese Respiratory Society
OR: Odds ratio
ROC: Receiver operating characteristic
RV: Residual volume
SEM: Standard error of the mean
