# Supplementary material for: Comprehensive analysis of allergen-specific IgE in COPD: mite-specific IgE specifically related to the diagnosis of asthma-COPD overlap
Source: Allergy Asthma Clin Immunol. 2021 Feb 4;17:13. doi: 10.1186/s13223-021-00514-9 (PMC7860183; doi:10.1186/s13223-021-00514-9)
Supplement: Supplementary file 1 — Additional file 1 Features of COPD or asthma described by the Japanese Respiratory Society Guideline [file 13223_2021_514_MOESM1_ESM.doc]

**Additional Files**

**Comprehensive analysis of allergen-specific IgE in COPD: Mite-specific IgE specifically related to diagnosis of asthma-COPD overlap**

**Authors:**

Hikaru Toyota, Naoya Sugimoto, Konomi Kobayashi, Yuki Suzuki, Ayaka Ito, Yuri Takeshita, Mariko Ujino, Fuminori Tomyo, Hirokazu Sakasegawa, Yuta Koizumi, Michio Kuramochi, Masao Yamaguchi, and Hiroyuki Nagase.

**Affiliation:**

Division of Respiratory Medicine and Allergology, Department of Medicine^1^, Teikyo University School of Medicine, Tokyo, Japan.

**Additional File 1. Features of COPD or asthma described by the Japanese Respiratory Society Guideline**

| Features of COPD  The presence of at least 1 feature | Features of asthma  The presence of at least 2 features |
| --- | --- |
| 1. Smoking history (10 pack-years or more)  or equivalent exposure to air pollution | 1. Variable or paroxysmal respiratory symptoms |
| 2. Emphysematous changes on chest CT | 2. A history of asthma before the age of 40 years |
| 3. Decreased gas exchange  (%D_LCO_ < 80% or %D_LCO_/V_A_ < 80%) | 3. FeNO > 35 ppb |
|  | 4-1) Comorbid perennial allergic rhinitis  4-2) Airway reversibility (FEV_1_ > 12% and > 200 mL)  4-3) Blood eosinophils > 5% or > 300 cells/µl  4-4) Elevated IgE level (total or allergen-specific IgE for perennial aeroallergen) |

**Notes:** ACO was defined as having fixed airflow limitation (FEV_1_:FVC<70%) after bronchodilator use with 1 or more of the COPD features plus 2 or more of the asthma features. Factor 4 in asthma is judged as positive when 2 or more criteria were fulfilled among 4-1 to 4-4 criterion.

**Abbreviations:** COPD, chronic obstructive pulmonary disease; DLco/V_A_, diffusing capacity of carbon monoxide/alveolar volume; FeNO, fraction of exhaled nitric oxide; FEV_1_, forced expiratory volume in 1 s; FVC, forced vital capacity; IgE, immunoglobulin E.

|  |  |  |  |
| --- | --- | --- | --- |
|  |  |  |  |
|  |  |  |  |
|  |  |  |  |
|  |  |  |  |
|  |  |  |  |
|  |  |  |  |
|  |  |  |  |
|  |  |  |  |
