# Supplementary material for: Comprehensive analysis of allergen-specific IgE in COPD: mite-specific IgE specifically related to the diagnosis of asthma-COPD overlap
Source: Allergy Asthma Clin Immunol. 2021 Feb 4;17:13. doi: 10.1186/s13223-021-00514-9 (PMC7860183; doi:10.1186/s13223-021-00514-9)
Supplement: Supplementary file 2 — Additional file 2 The number of patients analyzed in the study [file 13223_2021_514_MOESM2_ESM.docx]

**Additional File 2*.* The number of patients analyzed in the study**

COPD

85 patients

All features

analyzed

44 patients

Part of features analyzed

41 patients

Impossible to

diagnose ACO

9 patients

ACO 20 patients

Non-ACO 24 patients

ACO 14 patients

Non-ACO 18 patients

76 patients for ACO analysis

ACO 34 patients (44.7%)

Non-ACO COPD 42 patients (55.3%)

**Notes:** Asthma-chronic obstructive pulmonary disease (COPD) overlap (ACO) was diagnosed based on the ACO guideline of the Japanese Respiratory Society. All features for the diagnosis of ACO presented in Additional File 1 were analyzed in 44 patients, and 20 patients were diagnosed with ACO. Although full analysis was not conducted in 41 patients, the diagnosis of ACO was possible, except for 9 patients, and 14 patients were diagnosed with ACO. A total of 34 patients were diagnosed with ACO (44.7%).
