# Supplementary material for: Comprehensive analysis of allergen-specific IgE in COPD: mite-specific IgE specifically related to the diagnosis of asthma-COPD overlap
Source: Allergy Asthma Clin Immunol. 2021 Feb 4;17:13. doi: 10.1186/s13223-021-00514-9 (PMC7860183; doi:10.1186/s13223-021-00514-9)
Supplement: Supplementary file 3 — Additional file 3 Comparison of patient characteristics based on positivity for allergen specific IgE [file 13223_2021_514_MOESM3_ESM.docx]

| **Additional File 3. Comparison of patient characteristics based on positivity for allergen specific IgE** | | | | | | | | | | | | |
| --- | --- | --- | --- | --- | --- | --- | --- | --- | --- | --- | --- | --- |
|  | Moth | | Cockroach | | House dust | | *D. pteronyssinus* | | *Candida* | | Japanese cedar | |
|  | Positive | Negative | Positive | Negative | Positive | Negative | Positive | Negative | Positive | Negative | Positive | Negative |
| Subject (%) | 32.5 | 67.5 | 18.8 | 81.3 | 18.8 | 81.3 | 20.0 | 80.0 | 23.8 | 76.3 | 35.0 | 65.0 |
| Male (%) | 92.3* | 72.2 | 93.3 | 75.4 | 100.0* | 73.9 | 100.0* | 73.4 | 89.5 | 75.4 | 89.3 | 73.1 |
| Age (years) | 71.2 ± 1.4* | 75.0 ± 1.0 | 72.9 ± 1.9 | 73.9 ± 0.9 | 71.5 ± 1.9 | 74.3 ± 0.9 | 72.3 ± 1.8 | 74.1 ± 0.9 | 72.6 ± 1.7 | 74.1 ± 0.9 | 71.5 ± 1.3* | 75.0 ± 1.0 |
| Current smoker (%) | 23.1 | 5.6 | 13;3 | 10.8 | 13.3 | 10.8 | 18.8 | 9.4 | 21.1 | 8.2 | 10.7 | 11.5 |
| Smoking history (pack-year) | 59.9 ± 7.9 | 62.9 ± 5.5 | 63.3 ± 10.4 | 61.6 ± 5.0 | 54.3 ± 10.4 | 63.7 ± 5.0 | 58.8 ± 10.1 | 62.7 ± 5.0 | 67.5 ± 9.2 | 60.2 ± 5.1 | 57.5 ± 7.6 | 64.3 ± 5.6 |
| FVC, %predicted | 106.0±3.7 | 105.6±2.6 | 101.7±4.9 | 106.7 ± 2.3 | 105.6 ± 4.9 | 105.8 ± 2.4 | 105.7 ± 4.8 | 105.7±2.4 | 106.3 ± 4.4 | 105.6±2.4 | 106.1 ± 3.6 | 105.5 ± 2.6 |
| FEV_1_, %predicted | 73.0 ± 5.0 | 78.9 ± 3.5 | 69.5 ± 6.6 | 78.7 ± 3.2 | 72.1 ± 6.7 | 78.1 ± 3.2 | 75.6 ± 6.5 | 77.3 ± 3.2 | 68.8 ± 5.8 | 79.5 ± 3.3 | 76.4 ± 4.9 | 77.3 ± 3.6 |
| FEV_1_/FVC ratio (%) | 52.9 ± 2.7 | 55.4 ± 1.9 | 50.6 ± 3.6 | 55.5 ± 1.7 | 52.3 ± 3.6 | 55.1 ± 1.7 | 54.4 ± 3.5 | 54.6 ± 1.7 | 49.5 ± 3.1 | 56.2 ± 1.7 | 55.3 ± 2.6 | 54.2 ± 1.9 |
| RV, % predicted | 116.8 ± 6.4 | 112.0±4.4 | 136.7±9.7* | 110.2 ± 3.7 | 125.9 ± 9.4 | 111.5 ± 3.8 | 121.4 ± 9.0 | 112.1±3.9 | 121.6 ± 7.6 | 111.3±4.1 | 111.4 ± 6.0 | 114.8 ± 4.5 |
| DLco/V_A_, % predicted | 80.8 ± 6.6 | 73.6 ± 4.5 | 87.0 ± 10.4 | 74.3 ± 4.0 | 92.1 ± 9.6 | 73.2 ± 3.9 | 90.3 ± 9.2 | 73.2 ± 4.0 | 64.9 ± 7.8 | 79.0 ± 4.2 | 87.1 ± 5.9* | 69.4 ± 4.5 |
| Symptom of asthma (%) | 50.0 | 41.2 | 38.5 | 45.2 | 50.0 | 42.6 | 50.0 | 42.6 | 47.1 | 43.1 | 50.0 | 40.8 |
| History of asthma (%) | 20.8 | 31.4 | 23.1 | 29.0 | 21.4 | 29.5 | 21.4 | 29.5 | 29.4 | 27.6 | 26.9 | 28.6 |
| FeNO (ppb) | 26.7 ± 3.1 | 26.0 ± 2.3 | 31.2 ± 4.2 | 25.1 ± 2.0 | 29.6 ± 4.2 | 25.5 ± 2.0 | 28.5 ± 4.1 | 25.7 ± 2.0 | 26.2 ± 3.7 | 26.2 ± 2.1 | 25.8 ± 3.1 | 26.4 ± 2.3 |
| Perennial allergic rhinitis (%) | 44.0** | 5.9 | 42.9** | 12.9 | 66.7** | 6.6 | 66.7** | 6.6 | 38.9* | 12.1 | 22.2 | 16.3 |
| Airway reversibility positive (%) | 16.7 | 13.3 | 25.0 | 11.8 | 28.6 | 11.4 | 28.6 | 11.4 | 22.2 | 12.1 | 11.8 | 16.0 |
| Eosinophilia (%) | 38.5 | 37.0 | 13.3* | 43.1 | 26.7 | 40.0 | 31.3 | 39.1 | 42.1 | 36.1 | 46.4 | 32.7 |
| Eosinophil counts (cells/μl) | 229 ± 33 | 243 ± 22 | 183 ± 43 | 251 ± 20 | 244 ± 38 | 240 ± 20 | 253 ± 37 | 238 ± 20 | 262 ± 36 | 234 ± 21 | 265 ± 30 | 228 ± 22 |
| Serum total IgE (IU/ml) | 792±86** | 132 ± 59 | 878±122** | 221 ± 58 | 810±108** | 223 ± 58 | 857±101** | 198 ± 56 | 870±96** | 184 ± 55 | 646±87** | 186 ± 66 |
| **Notes:** *P* < 0.05*, *P* < 0.01** vs allergen-specific IgE negative group. Specific IgE was judged as positive when the class was equal to or greater than 1 by View39.  **Abbreviations:** DLco/VA, diffusing capacity of carbon monoxide/alveolar volume; FeNO, fraction of exhaled nitric oxide; FEV_1_, forced expiratory volume in 1 s; FVC, forced vital capacity; RV, residual volume; IgE, immunoglobulin E. | | | | | | | | | | | | |
