# Supplementary material for: Comprehensive analysis of allergen-specific IgE in COPD: mite-specific IgE specifically related to the diagnosis of asthma-COPD overlap
Source: Allergy Asthma Clin Immunol. 2021 Feb 4;17:13. doi: 10.1186/s13223-021-00514-9 (PMC7860183; doi:10.1186/s13223-021-00514-9)
Supplement: Supplementary file 4 — Additional file 4 Proportion of patients fulfilling the criteria for features of asthma [file 13223_2021_514_MOESM4_ESM.docx]

**Additional File 4. Proportion of patients fulfilling the criteria for features of asthma**

|  | ACO (%) | Non-ACO COPD (%) | *P* value |
| --- | --- | --- | --- |
| Variable or paroxysmal respiratory symptoms | 85.3 | 11.9 | <0.01** |
| A history of asthma before the age of 40 years | 61.8 | 9.5 | <0.01** |
| FeNO > 35 ppb ^a^ | 42.4 | 7.1 | <0.01** |
| More than 2 features of the following criteria | 65.6 | 26.3 | <0.01** |
| Comorbid perennial allergic rhinitis | 38.2 | 11.9 | <0.01** |
| Airway reversibility^b^ (*Δ*FEV_1_ > 12% and > 200 mL) | 23.8 | 8.3 | 0.15 |
| Blood eosinophils > 5% or > 300 cells/µl | 44.1 | 23.8 | 0.061 |
| Elevated IgE level  (Total or allergen-specific IgE for perennial aeroallergen) | 76.5 | 45.2 | <0.01** |

**Notes:** *P* < 0.05*, *P* < 0.01** between ACO and non-ACO COPD groups. The criterion for elevated IgE was judged as positive when the level of serum total IgE exceeded 100 IU/mL or the class of specific IgE was equal to or greater than class one by View39. FeNO^a^ (ACO n = 33, non-ACO COPD n = 42), airway reversibility^b^ (ACO n = 21, non-ACO COPD n = 24). **Abbreviations:** COPD, chronic obstructive pulmonary disease; FeNO, fraction of exhaled nitric oxide; FEV_1_, forced expiratory volume in 1 s; IgE, immunoglobulin E.
