# Supplementary material for: Comprehensive analysis of allergen-specific IgE in COPD: mite-specific IgE specifically related to the diagnosis of asthma-COPD overlap
Source: Allergy Asthma Clin Immunol. 2021 Feb 4;17:13. doi: 10.1186/s13223-021-00514-9 (PMC7860183; doi:10.1186/s13223-021-00514-9)
Supplement: Supplementary file 5 — Additional file 5 Relationships between blood eosinophil count, total IgE level, and FeNO [file 13223_2021_514_MOESM5_ESM.docx]

**Additional File 5. Relationships between blood eosinophil count, total IgE level, and FeNO**

*P* = 0.85

ρ = 0.027

*P* = 0.24

ρ = 0.14

*P* = 0.029*

ρ = 0.25

**Notes:** There were no significant relationships between biomarkers.

**Abbreviations:** FeNO, fraction of exhaled nitric oxide; IgE, immunoglobulin E.
